# The value of cervical length changes for the prediction of preterm birth with normal mid-trimester cervical length; a prospective longitudinal study

**DOI:** 10.3389/fmed.2026.1870436

**Published:** 2026-06-30

**Authors:** Sebile Guler Cekic, Ceren Unal, Can Benlioglu, M. Atay Ozten, Mert Turgal, Ebru Celik

**Affiliations:** 1 Department of Obstetrics and Gynecology, Koç University School of Medicine, Istanbul, Türkiye; 2 Perinatology Division, Department of Obstetrics and Gynecology, Koç University School of Medicine, Istanbul, Türkiye

**Keywords:** cervical length measurement, PPROM (preterm prelabor rupture of membranes), preterm (birth), prenatal ultrasonography, uterine cervical incompetence

## Abstract

**Introduction:**

Spontaneous preterm birth (sPTB) remains a leading cause of neonatal morbidity and mortality. Mid-trimester transvaginal cervical length (CL) measurement is an established screening tool, but it is unclear whether longitudinal change in CL between the 1st and 2nd trimesters (ΔCx) adds predictive value in women with a normal mid-trimester cervix.

**Methods:**

This prospective longitudinal cohort study included singleton pregnancies with 1st and 2nd trimester transvaginal CL measurements. Women with a normal mid-trimester cervix and no antenatal progesterone exposure comprised the primary low-risk cohort, while women with a short cervix were analyzed separately as an exploratory comparison cohort. CL was measured at 11–14 weeks (C1) and 18–24 weeks (C2). ΔCx and cervical slope were calculated. The primary outcome was sPTB <37 weeks. Additional analyses examined preterm prelabor rupture of membranes (PPROM) and PROM at term.

**Results:**

A total of 2,354 women had cervical length data in both trimesters; 2,292 constituted the primary low-risk cohort and 62 the exploratory short-cervix cohort. The overall rate of sPTB was 8.00 and 30.6% in the low-risk and short cervix cohorts, respectively (*p*<0.001). In the low-risk cohort, C1 (35.74 ± 4.61 mm vs. 35.50 ± 4.77 mm, *p* = 0.497) and C2 (37.44 ± 5.50 mm vs. 36.83 ± 5.55 mm, *p* = 0.148), ΔCx (1.69 ± 7.19 mm vs. 1.33 ± 6.81 mm, *p* = 0.515), and cervical slope were similar between term and preterm pregnancies (*p* = 0.558). In multivariable analysis, cervical slope was not associated with sPTB and did not improve discrimination beyond C2 and maternal characteristics. By contrast, the short-cervix cohort had markedly shorter C1 (30.31 ± 5.51 mm vs. 35.72 ± 4.62 mm, *p* < 0.001), C2 (21.61 ± 6.55 mm vs. 37.39 ± 5.50 mm, *p* **<** 0.001) a more negative cervical slope (–0.99 ± 1.00 mm vs. 0.19 ± 0.81 mm, *p* < 0.001). PPROM was more frequent (12.7% vs. 43.1%, *p* < 0.001) among preterm births in the low-risk cohort, and cervical slope was associated with PPROM (*p* = 0.030).

**Conclusion:**

In women with a normal mid-trimester cervix, longitudinal CL change does not improve prediction of sPTB beyond a single 2nd trimester measurement. The clinical value of cervical screening appears to lie primarily in identifying the short-cervix subgroup.

## 1 Introduction

Spontaneous preterm birth (sPTB) remains a major global health challenge and the leading cause of neonatal morbidity and mortality (1). The multifactorial etiology of PTB-encompassing inflammatory, infectious, vascular, and structural mechanisms-highlights the need for early identification of at-risk women to facilitate targeted preventive interventions. Transvaginal sonographic measurement of cervical length (CL) during the mid-trimester has been established as one of the most reliable and reproducible tools for predicting sPTB (2). A short cervix at the second trimester (CL < 25 mm) is strongly associated with an increased risk of sPTB (3). Preventive strategies, including vaginal progesterone treatment and cervical cerclage, have demonstrated efficacy in reducing preterm delivery rates within this high-risk subgroup (2, 4, 5).

However, population-based studies indicate that universal or risk-based CL screening alone has not substantially reduced the overall incidence of sPTB (6). In the United States, national statistics reveal that sPTB rates have remained relatively stable despite the widespread implementation of CL screening programs (7). In contrast, data from Israel demonstrated a modest decline -from 7.64 to 6.84%- following the introduction of routine CL measurements (4). These findings suggest that while CL screening identifies a subset of high-risk pregnancies, it may not identify all women at risk, highlighting the need for additional predictive markers that reflect the dynamic biological processes underlying cervical remodeling.

Emerging evidence supports the concept that cervical changes leading to preterm labor may begin earlier in gestation and progress gradually, even in women with an apparently normal mid-trimester CL (8–10). Therefore, *longitudinal assessment of CL*, incorporating both 1st and 2nd trimester measurements, may provide enhanced predictive power by identifying subtle trends in cervical shortening before critical thresholds are reached. To test this hypothesis, we conducted a *prospective longitudinal study* in asymptomatic singleton pregnancies with a normal cervix (CL > 25 mm) at the mid-trimester scan. We evaluated the change in CL between 1st and 2nd trimester assessments and examined whether the degree of cervical shortening over time is independently associated with the risk of sPTB ( < 37 weeks). By focusing on women without an overtly short cervix, our study aims to refine risk stratification and inform more personalized preventive strategies in routine obstetric practice.

## 2 Materials and methods

### 2.1 Study design and population

This prospective longitudinal study was conducted between May 2019 and February 2024 to investigate the predictive value of CL changes for sPTB among women with a normal CL. The study protocol was reviewed and approved by the Koç University Institutional Ethics Committee (2019.093IRB2.030). All procedures adhered to institutional standards and the principles of the Declaration of Helsinki.

Women with singleton pregnancies were invited to participate and assessed for eligibility during the first trimester (11+0 to 13+6 weeks of gestation) after providing written informed consent. Final recruitment and enrollment were completed in the second trimester. Maternal demographic and clinical data including age, body mass index (BMI), parity, obstetric and gynecologic history, pregnancy outcomes and smoking status were obtained from the hospital’s electronic medical record system.

Women were excluded from the primary low-risk cohort if CL data were missing from either trimester assessment; if they underwent cerclage or received progesterone therapy (except for IVF patients during the first trimester); if they had multiple gestation, fetal chromosomal abnormalities, or structural anomalies; experienced pregnancy termination; had preterm birth secondary to preeclampsia or fetal growth restriction; or were lost to follow-up prior to delivery. Women with a short cervix ( ≤ 25 mm) were excluded from the primary low-risk analysis but were retained as a separate exploratory comparison cohort. Treatment data for the short cervix group, including cerclage and vaginal progesterone use, were obtained from hospital records.

Gestational age (GA) was initially determined based on the maternal last menstrual period and subsequently confirmed by first-trimester ultrasound, using the measurement of fetal crown–rump length for dating accuracy.

### 2.2 Cervical length measurement

CL was measured by transvaginal ultrasound by two experienced sonographers (E.C. and M.T.) in accordance with the standardized protocol of the International Society of Ultrasound in Obstetrics and Gynecology (ISUOG) to ensure methodological consistency and reproducibility (11). Following bladder emptying, each participant was positioned in the lithotomy position, and a mid-sagittal view of the endocervical canal was obtained using a Voluson E8 Expert ultrasound system (GE Healthcare, Zipf, Austria GmbH & Co OG) equipped with a 4–9 MHz transvaginal transducer. The image was optimized to visualize the entire length of the cervix, extending from the external os through the endocervical canal to the internal os, with care taken to avoid undue probe pressure on the cervix.

Each examination lasted approximately 3 min. For each patient, three consecutive measurements of CL were obtained, and the shortest measurement between the internal and external os was recorded for analysis.

### 2.3 Pregnancy outcomes

Pregnancy outcomes including GA at birth, mode of delivery, birth weight and the occurrence of preterm prelabor rupture of membranes (PPROM) or prelabor rupture of membranes (PROM) were retrieved from the institutional electronic medical record system for patients who delivered at our center. PROM/PPROM was recorded when labor began with rupture of membranes rather than with active uterine contractions. For pregnant women whose delivery data were not available in the hospital database, follow-up information was obtained through telephone contact. For the membrane-rupture phenotype regression analyses, only deliveries with complete PROM/PPROM documentation and complete covariate and cervical-slope data were included; deliveries with missing membrane-rupture documentation were therefore excluded from these comparisons, leaving 1,761 term births and 160 preterm births for the respective phenotype analyses.

### 2.4 Statistical analysis

All analyses were performed using R (R Foundation for Statistical Computing, Vienna, Austria; version 4.4.1) with the packages *dplyr*, *tableone*, *broom*, and *pROC*. A two-sided *p* < 0.05 was considered statistically significant. Maternal and neonatal characteristics, as well as cervical measurements, were summarized separately for pregnancies resulting in term and sPTB ( < 37+0 weeks). Continuous variables (e.g., GA at birth, birthweight, BMI, CL) were described as mean (standard deviation) or median (interquartile range), according to distribution, and compared between groups using the Student’s *t*-test or Mann-Whitney U test as appropriate. Categorical variables (e.g., parity, smoking status, mode of delivery) were presented as number (percentage) and compared using the χ^2^ test or Fisher’s exact test. For each pregnancy, first trimester CL (C1) and mid-trimester CL (C2) were obtained from transvaginal ultrasound examinations. GA at each scan was recorded in weeks and days and converted to decimal weeks. The rate of cervical change between scans (rate of cervical change) was calculated as the difference in cervical length divided by the time interval between examinations (positive slope values denote net cervical lengthening and negative values denote net shortening between the first- and second-trimester scans) expressed as millimeters per week:


$$\mathrm{slope}\,\left(\mathrm{mm}/\mathrm{week}\right)=\frac{C\,2-C\,1}{{\text{GA}}_{2}-{\text{GA}}_{1}}$$

For slope-based analyses, pregnancies were excluded if the interval between the two scans was zero or negative (5 cases with same-day or reversed-order scans). An additional 5 pregnancies had missing interval data and were also excluded. Four further pregnancies with extreme outlying slope values were excluded after visual inspection of the slope distribution (slopes ranging from -9.80 to +12.6 mm/week, compared with a sample range of –2.82 to +3.44 mm/week in the analytic cohort), leaving 2,317 pregnancies for slope analyses prior to further eligibility filtering. To explore whether the impact of the rate of cervical change differed according to the underlying C2, we fitted an additional model including an interaction term between C2 and rate of cervical change (C2 × slope).

We fitted three nested models: (i) a base model including maternal age, BMI and smoking; (ii) a model additionally including C2; and (iii) a model further including rate of cervical change (mm/week). Adjusted odds ratios (aORs) with 95% confidence intervals (CIs) were reported for each predictor. Incremental predictive value of rate of cervical change beyond C2 was assessed by comparing models (ii) and (iii) using the likelihood-ratio test and by examining changes in model deviance, Akaike information criterion (AIC) and the area under the receiver-operating characteristic curve (c-statistic). All regression analyses were conducted using complete cases; women with missing data for outcome, cervical measurements, GA at either scan or any covariate were excluded from the respective models.

## 3 Results

**FIGURE 1:**
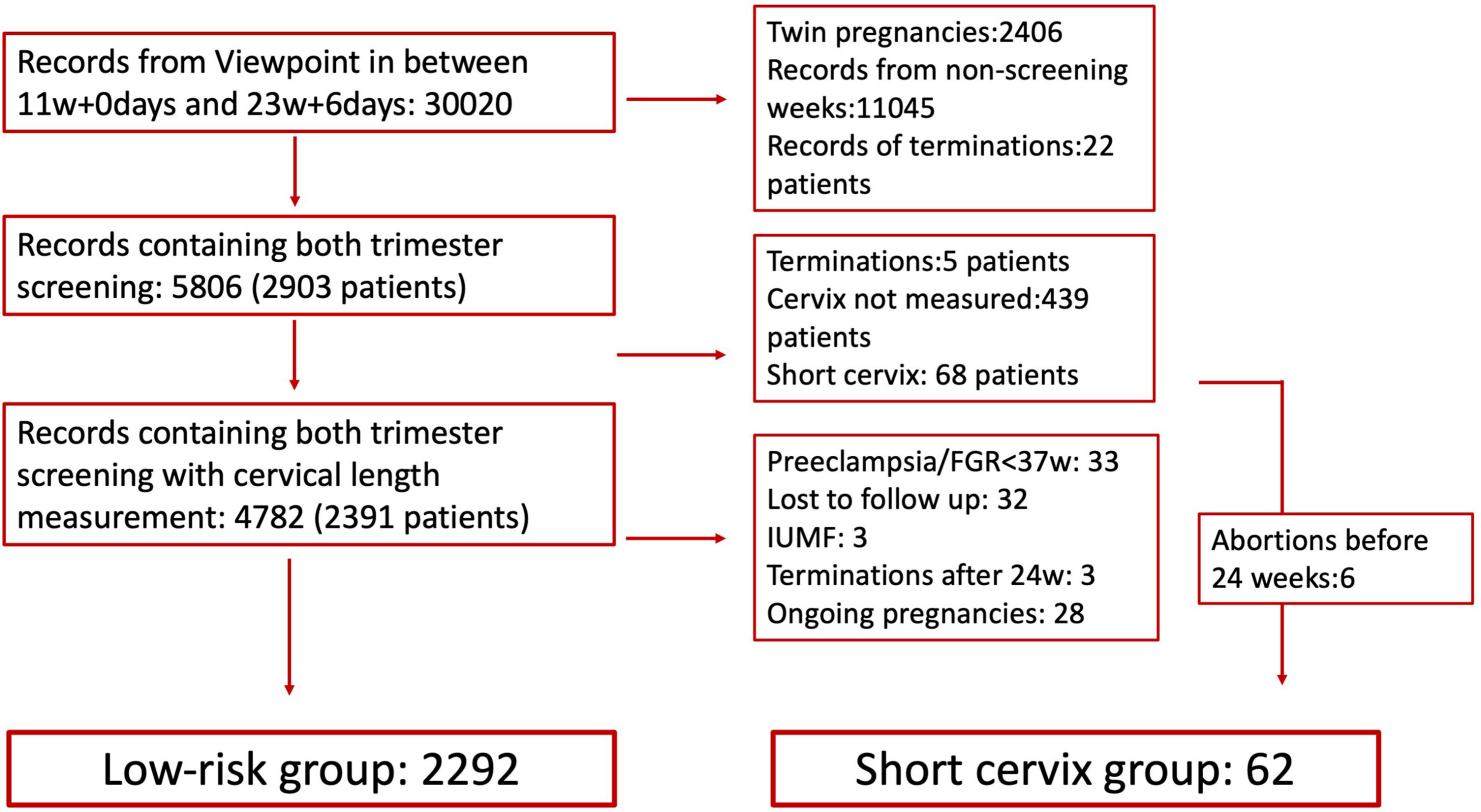
Flow chart of the patients included in the study.

**FIGURE 2:**
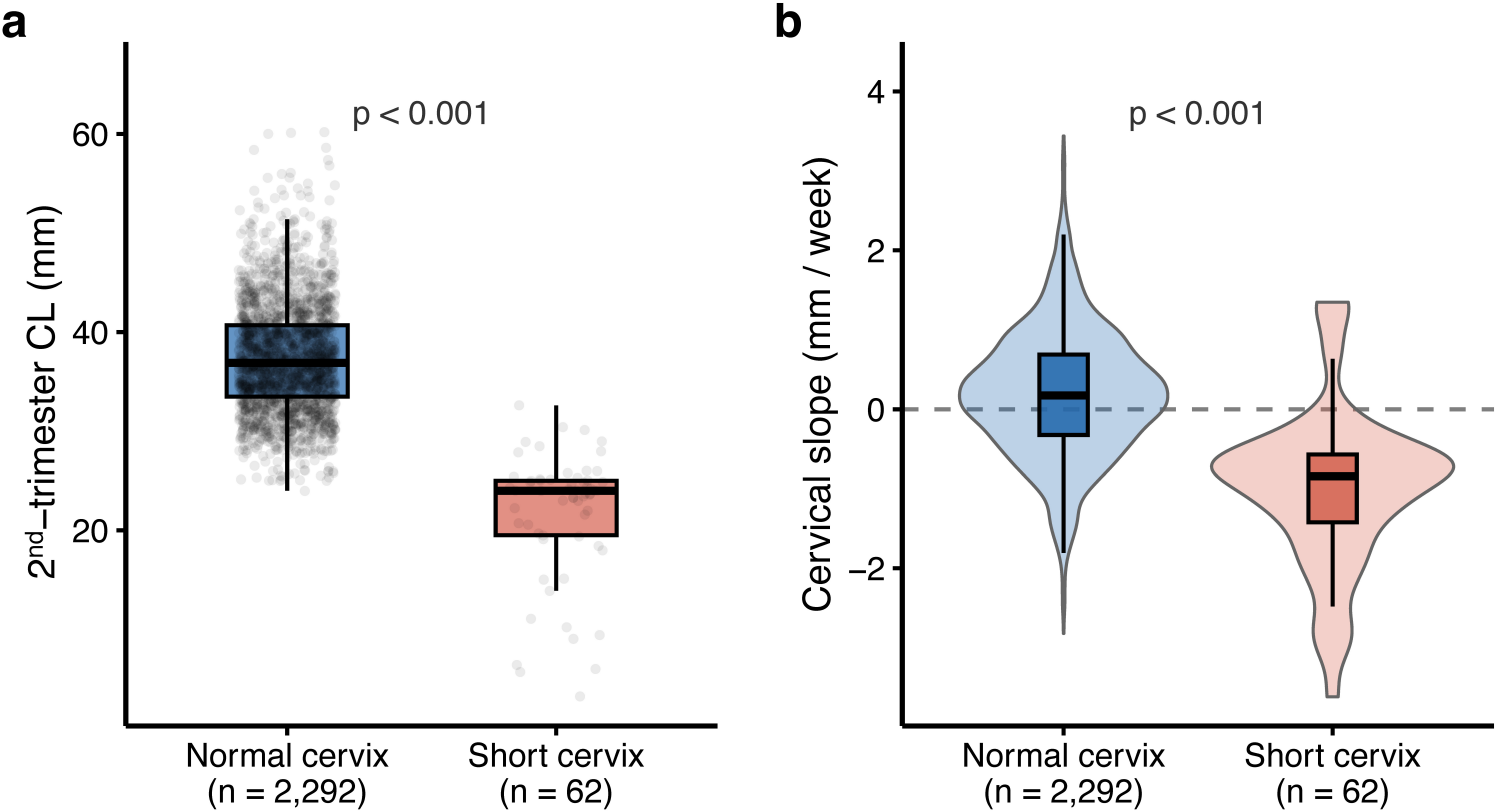
Mean cervical length in the second trimester and cervical slope change (low-risk cohort vs. short cervix cohort). Comparison of 2^nd^ trimester cervical length (CL) and cervical slope between the study cohort and the exploratory short cervix cohort. **(a)** Second-trimester cervical length (mm), and **(b)** cervical slope (mm/week). Women in the short cervix cohort had significantly shorter 2^nd^ trimester CL and a more negative cervical slope than women in the study cohort (*p* < 0.0001).

A flow chart of the cohort is shown in Figure 1. A total of 2,354 women had cervical length measurements available in both trimesters. Of these, 2,292 women with a normal mid-trimester cervix and no antenatal progesterone exposure constituted the primary low-risk cohort, whereas 62 women with a short cervix comprised an exploratory comparison cohort. Within the short cervix group, 26 women (41.9%) underwent cerclage, whereas the remaining women received vaginal progesterone. Women in the short-cervix cohort had significantly shorter C1 and C2, a more negative rate of cervical change, lower GA at delivery, and lower birthweight than women in the low-risk cohort (*p* < 0.001, Figure 2 and Table 1). Preterm birth was also markedly more frequent in the short-cervix cohort (30.6% vs. 8.0%, *p* < 0.001, Table 1), and smoking was more common (16.4% vs. 6.9%, *p* = 0.016, Table 1). In contrast, age, BMI, GA at cervical assessment, and parity were similar between cohorts (*p* > 0.05, Table 1).

**TABLE 1:** Demographic and obstetric properties of patients.

Variable	Normal cervix (*n* = 2,292)	Short cervix (*n* = 62)	*p-*value
Maternal age (years)	31.75 (4.79)	32.06 (4.90)	0.619
BMI (kg/m^2^)	24.64 (4.16)	24.51 (5.19)	0.819
1st trimester cervical length (mm)	35.72 (4.62)	30.31 (5.51)	**< 0.001**
Gestational age of 1st trimester cervical length measurement	12.43 (0.66)	12.44 (0.75)	0.992
2nd trimester cervical length (mm)	37.39 (5.50)	21.61 (6.55)	**< 0.001**
Gestational age of 2nd trimester cervical length measurement	21.39 (0.93)	21.37 (0.97)	0.883
Rate of cervical change (mm/week)	0.19 (0.81)	–0.99 (1.00)	**< 0.001**
ΔCx (mm)	1.66 (7.16)	-8.90 (8.43)	**< 0.001**
Gestational age of birth	38.61 (1.40)	36.74 (3.39)	**< 0.001**
Fetal weight (g)	3287.16 (464.20)	2987.91 (671.62)	**< 0.001**
Multiparity	901 (39.3%)	12 (42.9%)	0.851
Smoking (yes)*	156 (6.9%)	9 (16.4%)	**0.016**
Mode of delivery (cesarean)	1424 (66.9%)	33 (60%)	0.313
Preterm birth	183 (8.0%)	19 (30.6%)	**< 0.001**
PROM/PPROM*	347 (15.1%)	20 (32.3%)	**0.001**

Values are presented as mean (standard deviation) for continuous variables and number/total (percentage) for categorical variables.

*Total n differs slightly across variables due to missing data (denominators shown in the categorical rows). BMI, Body mass index; ΔCx, Cervical length difference between first and second trimester cervical length measurements; PROM, Prelabor rupture of membranes; PPROM, Preterm prelabor rupture of membranes. Values reaching statistical significance are presented in bold.

**FIGURE 3:**
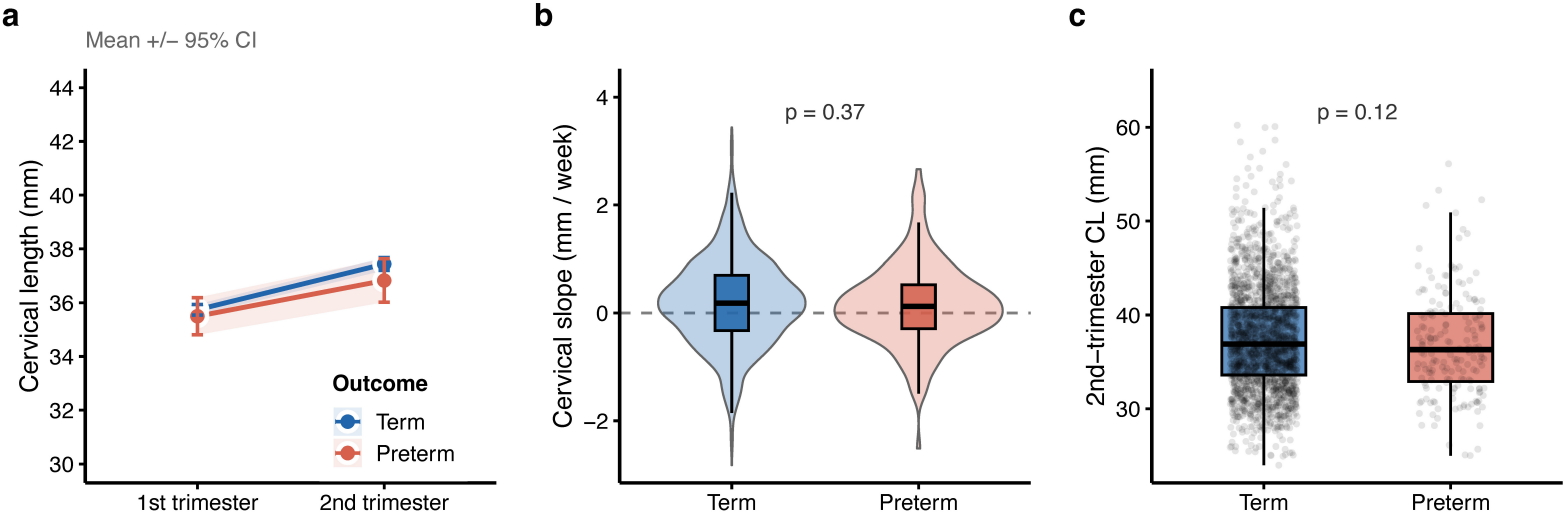
Cervical dynamics of low-risk cohort. Cervical dynamics in the progesterone-naive study cohort according to pregnancy outcome. Full cohort (*n* = 2,292). **(a)** Mean cervical length (CL) in the 1^st^ and 2^nd^ trimesters in term and preterm pregnancies, with 95% confidence intervals. **(b)** The distribution of cervical slope (mm/week), and **(c)** the distribution of 2^nd^ trimester CL (mm). 1^st^ and 2^nd^ trimester CL, as well as cervical slope, were similar between term and preterm births, with substantial overlap between groups.

Within the primary low-risk cohort (*n* = 2,292), 2,109 (92.0%) women delivered at term and 183 (8.0%) delivered preterm (Table 2). Maternal age, BMI, parity, smoking status, and GA at 1st and 2nd trimester cervical assessment did not differ significantly between the term and preterm groups (*p* > 0.05, Table 2). C1 was similar in term and preterm pregnancies (35.74 ± 4.61 mm vs. 35.50 ± 4.77 mm, *p* = 0.497; Figure 3a), as was C2 (37.44 ± 5.50 mm vs. 36.83 ± 5.55 mm, *p* = 0.148; Figure 3c). Likewise, rate of cervical change (0.19 ± 0.81 vs. 0.15 ± 0.76 mm/week, *p* = 0.558) did not differ significantly between groups (Figure 3b). CL increased between scans in both groups. Mean ΔCx was 1.68 ± 7.18 mm in term and 1.29 ± 6.71 mm in sPTB (*p* = 0.44, Table 2). GA at birth and fetal weight were significantly lower in the preterm group, and cesarean delivery and PPROM were more frequent (*p* < 0.001, Table 2).

**TABLE 2:** Demographic and obstetric properties of low-risk patients compared with preterm birth.

Variable	Term (*n* = 2109)	Preterm (*n* = 183)	*p* value
Maternal age (years)	31.72 (4.79)	32.13 (4.78)	0.266
BMI (kg/m^2^)	24.61 (4.16)	25.04 (4.15)	0.198
1st trimester cervical length (mm)	35.74 (4.61)	35.50 (4.77)	0.497
Gestational age of 1st trimester cervical length measurement	12.43 (0.66)	12.43 (0.64)	0.997
2nd trimester cervical length (mm)	37.44 (5.50)	36.83 (5.55)	0.148
Gestational age of 2nd trimester cervical length measurement	21.38 (0.92)	21.48 (1.01)	0.183
Rate of cervical change (mm/week)	0.19 (0.81)	0.15 (0.76)	0.558
ΔCx (mm)	1.69 (7.19)	1.33 (6.81)	0.515
Gestational age of birth	38.88 (0.95)	35.49 (1.92)	**< 0.001**
Fetal weight (gr)	3338.87 (422.98)	2731.80 (522.10)	**< 0.001**
Multiparity	821 (38.9%)	80 (43.7%)	0.233
Smoking (yes)*	141 (6.8%)	15 (8.4%)	0.522
Mode of delivery (cesarean)	1286 (67.8%)	138 (82.1%)	**< 0.001**
PROM/PPROM (%)*	268 (12.7%)	79 (43.1%)	**< 0.001**

Values are presented as mean (standard deviation) for continuous variables and number/total (percentage) for categorical variables.

*Total n differs slightly across variables due to missing data (denominators shown in the categorical rows). BMI, Body mass index; ΔCx, Cervical length difference between first and second trimester cervical length measurements; PROM, Prelabor rupture of membranes; PPROM, Preterm prelabor rupture of membranes. Values reaching statistical significance are presented in bold.

Because PPROM was significantly more frequent among preterm births, we performed subgroup analyses for PPROM in preterm births and PROM in term births (Supplementary Figure 1 and Supplementary Table 1). Among preterm births, women with PPROM (*n* = 69) and those without PPROM (*n* = 91) had comparable maternal age, BMI, 1st and 2nd trimester cervical lengths, GA at cervical assessment, cervical slope, and ΔCx (*p* > 0.05, Supplementary Table 1). However, the PPROM group delivered at an earlier GA (35.13 ± 2.13 vs. 35.78 ± 1.75 weeks, *p* = 0.035) and had lower birthweight (2608.00 ± 452.51 vs. 2831.12 ± 555.42 g, *p* = 0.011). Cesarean delivery was less common in the PPROM group (73.9% vs. 89.0%, *p* = 0.023). Similar findings were found among term births, PROM occurred in 220 women, whereas 1,541 term births occurred without PROM. Cesarean delivery was substantially less frequent in the PROM group (47.7% vs. 71.9%, *p* < 0.001).

Across outcomes, C1, C2, and cervical slope were not associated with preterm birth or PROM at term, and predictive performance was poor (AUC 0.561 and 0.549, respectively). For PPROM, C1 showed a borderline association (*p* = 0.055), C2 was not significant, and cervical slope was significantly associated with the outcome (*p* = 0.030), although discrimination remained modest (AUC 0.664) (Supplementary Figure 2 and Supplementary Table 2).

**FIGURE 4:**
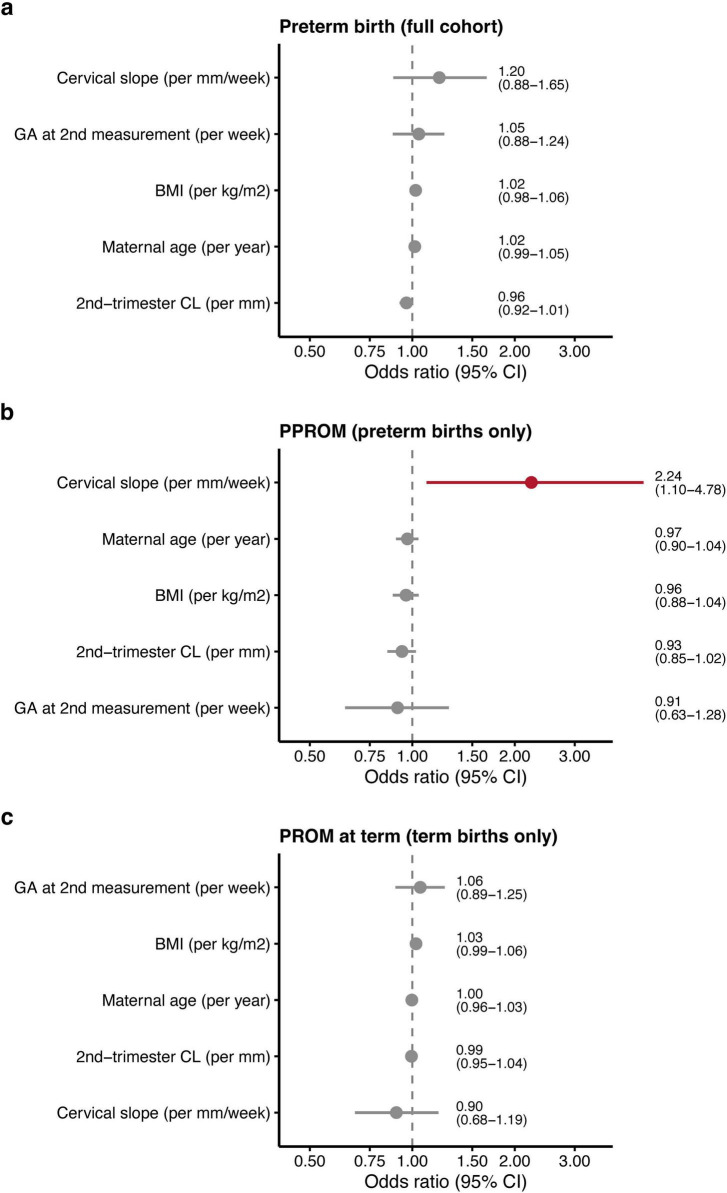
Forest plots of adjusted odds ratios (ORs) with 95% confidence intervals from multivariable logistic regression models in the low-risk cohort. **(a)** Predictors of spontaneous preterm birth in the low-risk cohort, **(b)** predictors of preterm prelabor rupture of membranes (PPROM) among preterm births, and **(c)** predictors of prelabor rupture of membranes (PROM) at term. Models included maternal age, body mass index, gestational age at 2^nd^ measurement, 2^nd^ trimester cervical length, and cervical slope. The x-axis is presented on a logarithmic scale. Colored estimates indicate statistically significant associations. Smoking retained in all models but excluded from figure (wide CIs). Log scale; colored = *p* < 0.05.

In multivariable analysis for preterm birth, maternal age, BMI, smoking, GA at second measurement, C2, and cervical slope were not significantly associated with outcome (Figure 4 and Table 3). C2 showed a weak inverse association that did not reach statistical significance (OR 0.96, 95% CI 0.92–1.01; *p* = 0.089), while cervical slope was not predictive (OR 1.20, 95% CI 0.88–1.65; *p* = 0.257) (Figure 4a and Table 3). In the model comparing PPROM with non-PPROM among preterm births, a more positive cervical slope (greater apparent cervical lengthening between the first- and second-trimester scans) was the only variable significantly associated with the PPROM phenotype (OR 2.24, 95% CI 1.10–4.78; *p* = 0.030), whereas C2 was not (OR 0.93, 95% CI 0.85–1.02; *p* = 0.153) (Table 4 and Figure 4b). In contrast, neither C2 (OR 0.99, 95% CI 0.95–1.04; *p* = 0.799) nor cervical slope (OR 0.90, 95% CI 0.68–1.19; *p* = 0.454) was associated with PROM at term (Figure 4c; Supplementary Figure 1; and Supplementary Table 3). Smoking showed a borderline inverse association (OR 0.51, 95% CI 0.22–1.00; *p* = 0.072), but this did not reach statistical significance (Supplementary Table 3).

**TABLE 3:** Adjusted ORs for preterm birth in low-risk cohort.

Variable	OR (95% CI)	*p-*value
Maternal age (per year)	1.02 (0.99–1.05)	0.252
BMI (per kg/m^2^)	1.02 (0.98–1.06)	0.233
Smoking (yes vs. no)	1.08 (0.55–1.92)	0.811
GA at 2nd measurement (per week)	1.05 (0.88–1.24)	0.618
2nd trimester CL (per mm)	0.96 (0.92–1.01)	0.089
Cervical slope (per mm/week)	1.20 (0.88–1.65)	0.257

OR, Odds ratio; BMI, Body mass index; GA, Gestational age; CL, Cervical length.

**TABLE 4:** Adjusted ORs for PPROM versus non-PPROM among preterm births in the low-risk cohort.

Variable	OR (95% CI)	*p-*value
Maternal age (per year)	0.97 (0.90–1.04)	0.387
BMI (per kg/m^2^)	0.96 (0.88–1.04)	0.340
Smoking (yes vs. no)	0.38 (0.05–1.80)	0.255
GA at 2nd measurement (per week)	0.91 (0.63–1.28)	0.574
2nd-trimester CL (per mm)	0.93 (0.85–1.02)	0.153
Cervical slope (per mm/week)	2.24 (1.10–4.78)	**0.030**

OR, Odds ratio; BMI, Body mass index; GA, Gestational age; CL, Cervical length. Values reaching statistical significance are presented in bold.

ROC analysis showed that adding cervical slope to 2nd trimester cervical length did not improve discrimination for preterm birth (AUC 0.555 vs. 0.561; DeLong *p* = 0.587) or PROM at term (AUC 0.546 vs. 0.549; DeLong *p* = 0.785). For PPROM, the AUC increased from 0.588 to 0.664, suggesting modest improvement, although the difference did not reach statistical significance (DeLong *p* = 0.095) (Supplementary Figure 2).

In a sensitivity analysis including progesterone users, antenatal progesterone use was strongly associated with preterm birth (OR 5.93, 95% CI 2.33–14.03; *p* < 0.001), whereas 2nd trimester cervical length and cervical slope remained non-significant predictors (Supplementary Table 4).

Within the exploratory short-cervix cohort, neither C1 and C2 nor cervical slope was significantly associated with preterm birth (Supplementary Table 5).

## 4 Discussion

In this prospective longitudinal cohort study, we evaluated whether CL progression between the first and second trimesters improves prediction of sPTB. In the primary low-risk cohort of women with a normal mid-trimester cervix, 1st and 2nd trimester CL, ΔCx, and cervical slope were all similar between term and preterm pregnancies. In multivariable analysis, cervical slope was not independently associated with sPTB, and adding this longitudinal parameter did not improve model fit or discrimination beyond 2nd trimester CL and maternal characteristics. Maternal age, BMI, and smoking were likewise not associated with sPTB in this low-risk cohort. By contrast, the exploratory short-cervix cohort showed markedly shorter CL values, a more negative cervical slope, and a substantially higher preterm birth rate, emphasizing the clinical importance of the short-cervix threshold. Although longitudinal CL change did not improve prediction of sPTB overall, PPROM was significantly more frequent among preterm births, and cervical slope was associated with PPROM, suggesting that dynamic cervical change may be relevant to membrane-related pathways.

Prior studies in low-risk populations report a mean 1st trimester CL of approximately 43–46 mm, supporting its feasibility as an early screening parameter (12, 13). Souka et al. further showed that CL measured at 11–14 weeks can anticipate shorter CL later in pregnancy and thereby inform risk of preterm birth (12). In our low-risk cohort, mean 1st trimester CL was 35.7 mm (SD∼4.6 mm) in women who delivered at term and 35.4 mm (SD∼4.7 mm) in those with sPTB; corresponding 2nd trimester values were 37.4 mm (SD∼5.5 mm) and 36.7 mm (SD∼5.5 mm), respectively. Although CL increased between the first and second trimester in both groups, the ΔCx was smaller but not significant in sPTB group (approximately +1.7 mm vs. +1.3 mm, *p* = 0.44). When we modeled rate of cervical change (mm/week) as a longitudinal measure, it was not significantly associated with sPTB after adjustment for maternal age, BMI, smoking and C2 (aOR ∼1.20 per 1 mm/week, 95% CI 0.88–1.63, *p*≈0.25), and its inclusion did not improve model performance on likelihood-ratio testing. C2 showed only a borderline inverse association with sPTB (aOR ∼0.96 per mm, 95% CI 0.92–1.00, *p*≈0.06), with a modest effect size. Taken together, these findings suggest that, among women with a normal mid-trimester cervix, early CL measurement and serial change do not add meaningful predictive value beyond a single mid-trimester assessment.

Importantly, previous studies have shown that combining cervical length (CL) with maternal characteristics, particularly obstetric history, improves prediction of early spontaneous preterm birth, with reported detection rates of up to 54.8% at a 10% false-positive rate (14, 15). In our analysis, however, maternal age, BMI, and smoking status were not independently associated with sPTB and did not improve predictive performance when added to CL in the low-risk cohort. This likely reflects the relatively homogeneous risk profile of women with a normal mid-trimester cervix. In contrast, smoking was significantly more frequent in the exploratory short-cervix cohort, suggesting that maternal risk factors may be more relevant in women who already exhibit cervical shortening than in those whose cervical length remains within the normal range.

The universal application of CL screening remains debated, particularly in low-risk populations in whom the baseline incidence of preterm birth is modest (16–18). Nevertheless, early identification of a short cervix remains clinically important, as vaginal progesterone has been shown to reduce preterm birth in this high-risk subgroup (3, 19, 20). In the present study, women with a normal mid-trimester cervix had a relatively low rate of early preterm birth, whereas the exploratory short-cervix cohort showed markedly higher rates of preterm birth together with more pronounced cervical shortening dynamics. These findings support the concept that the clinical value of CL screening lies primarily in identifying women below the short-cervix threshold, in whom targeted interventions may be offered, rather than in serial monitoring of women whose cervical length remains within the normal range.

We also explored the clinical relevance of CL trajectory. On average, CL increased from the first to the second trimester in both outcome groups, and neither the ΔCx nor the rate of change differed significantly between term and preterm deliveries. Mean CL values at the mid-trimester scan were slightly higher than at the first-trimester scan (mean difference approximately 1.6 mm), a difference that is small and likely within the range of expected measurement variability. As shown in previous studies (21, 22), the “elongation” of the cervix over time may partly reflect technical and physiological factors. First-trimester CL measurement is more challenging because of the natural cervical curvature, and the relative position of the uterus, which can lead to underestimation (11). As gestation advances, the cervix tends to be straight, so 2nd trimester measurements may appear longer even without true anatomical change. Yet, a modest physiological increase in CL with uterine growth cannot be excluded. In our multivariable analysis, however, rate of cervical change was not predictive of sPTB, and models including slope did not outperform those based on C2 alone. These findings align with prior studies reporting that, in women without a short cervix and without prophylactic intervention, there is a moderate increase in CL from first to second trimester (21, 22). Interestingly, we found that an increased cervical slope was associated with PPROM, suggesting that dynamic cervical change may be relevant to membrane-related pathways. Given the small number of PPROM events (*n* = 69) and the wide confidence interval, this finding should be interpreted with caution and may partly reflect first-trimester measurement variability rather than a true biological effect.

PPROM also warrants attention. In this cohort, PPROM was significantly more frequent among women who delivered preterm. Prediction of PPROM remains challenging. Rode et al. proposed a model incorporating first-trimester CL ≤ 25 mm, low PAPP-A, nulliparity and prior cervical conization as risk factors (23); of these, only nulliparity was common in this cohort. Emerging evidence suggests that cervical and vaginal microbiota influence membrane integrity and inflammatory activation: women who develop PPROM demonstrate a shift toward a pro-inflammatory vaginal immune profile (24), and disruption of a *Lactobacillus*-dominant flora has been associated with increased expression of matrix metalloproteinases and pro-inflammatory cytokines, potentially contributing to early membrane rupture (25, 26). Future studies integrating CL assessment with microbiota profiling and inflammatory biomarkers may improve risk stratification for PPROM (27).

Our results suggest that, in low-risk women with normal CL, routine serial CL monitoring is unlikely to be beneficial and repeat scans should be reserved for women with additional risk factors or borderline CL values. A systematic review similarly concluded that change in transvaginal CL over time is not a clinically useful test for predicting preterm birth in singleton or twin gestations (28). Collectively, the available evidence supports *a single mid-trimester (18–22 weeks) CL measurement* as the most reliable screening time point (1, 29–31).

This study has several strengths, including its prospective design, relatively large sample size, and the use of standardized transvaginal sonographic techniques performed by experienced operators. By focusing on a clinically relevant low-risk group and excluding women receiving progesterone or cerclage from the primary analysis, we minimized confounding by indication and treatment. The availability of both 1st and 2nd trimester scans also allowed estimation of cervical change over an approximately 8–9-week interval.

Nevertheless, some limitations should be acknowledged. Exclusion of women with a short cervix and those receiving intervention reduced the overall event rate and may have limited our power to detect small, yet potentially clinically relevant, associations. CL has a nonlinear relationship with sPTB, with risk increasing steeply below the 25 mm threshold and attenuating above this value; therefore, the limited predictive ability of CL-related parameters in our cohort was not unexpected. Importantly, this does not diminish the clinical value of mid-trimester CL screening for identifying the high-risk short-cervix subgroup in whom targeted interventions are most effective. The exploratory short-cervix cohort received treatment, and comparisons with the untreated low-risk cohort should therefore be interpreted cautiously, as observed differences may reflect both baseline risk and intervention effects. In addition, our analysis was based on complete cases, and although the amount of missing data was modest, some selection bias cannot be excluded. The PPROM analysis was a within-preterm-birth comparison distinguishing PPROM from non-PPROM deliveries, not an antenatal prediction model in the whole at-risk cohort; the slope-PPROM association would require prospective validation before any clinical screening application. We also did not include biochemical, inflammatory, or microbiome markers, which may improve predictive accuracy when combined with sonographic measures. Finally, this was a single-center study, and the findings may not be generalizable to higher-risk populations or to settings with different screening and treatment practices.

## 5 Conclusion

In women with a normal mid-trimester cervical length, longitudinal change in cervical length between the first and second trimesters does not improve prediction of spontaneous preterm birth beyond a single second-trimester measurement. These findings suggest that routine serial cervical length monitoring is not warranted in low-risk women with a normal cervix and support the use of a single mid-trimester transvaginal cervical length assessment, with targeted interventions and closer surveillance reserved for women with a short cervix or additional risk factors. Because current methods remain limited in predicting PPROM, further studies integrating cervical imaging with microbiome and immunological biomarkers are needed to improve PPROM risk stratification.

## Data Availability

The original contributions presented in the study are included in the article/Supplementary material, further inquiries can be directed to the corresponding author.
