# Supplementary material for: The value of cervical length changes for the prediction of preterm birth with normal mid-trimester cervical length; a prospective longitudinal study
Source: Front Med (Lausanne). 2026 Jun 30;13:1870436. doi: 10.3389/fmed.2026.1870436 (PMC13365343; doi:10.3389/fmed.2026.1870436)
Supplement: Supplementary file 1 [file Table_1.DOCX]

Supplementary Material

# Supplementary Tables

**Supplementary Table 1. Demographic and obstetric properties of term births of low-risk patients complicated with prelabor rupture of membranes**

| **Variable** | **No PROM (n=1541)** | **PROM (n=220)** | ***p* value** |
| --- | --- | --- | --- |
| Maternal age (years) | 31.73 (4.90) | 31.60 (4.22) | 0.703 |
| BMI (kg/m²) | 24.53 (4.12) | 24.95 (4.54) | 0.185 |
| 1^st^ trimester cervical length (mm) | 35.71 (4.63) | 35.90 (4.63) | 0.567 |
| Gestational age of 1^st^ trimester cervical length measurement | 12.42 (0.64) | 12.46 (0.58) | 0.366 |
| 2^nd^ trimester cervical length (mm) | 37.44 (5.53) | 37.06 (5.23) | 0.338 |
| Gestational age of 2^nd^ trimester cervical length measurement | 21.35 (0.90) | 21.39 (0.89) | 0.539 |
| Rate of cervical change (mm/week) | 0.20 (0.82) | 0.12 (0.80) | 0.227 |
| ΔCx (mm) | 1.73 (7.23) | 1.16 (6.95) | 0.279 |
| Gestational age of birth | 38.90 (0.93) | 38.58 (0.94) | **<0.001** |
| Fetal weight (gr) | 3352.42 (426.79) | 3252.82 (383.15) | **0.003** |
| Multiparity | 606 (39.3%) | 73 (33.2%) | 0.094 |
| Smoking (yes) | 114 (7.5%) | 10 (4.6%) | 0.151 |
| Mode of delivery (caesarean) | 1105 (71.9%) | 105 (47.7%) | **<0.001** |

Values are presented as mean (standard deviation) for continuous variables and number/total (percentage) for categorical variables.

*Total n differs slightly across variables due to missing data (denominators shown in the categorical rows).

BMI: Body mass index

ΔCx: Cervical length difference between first and second trimester cervical length measurements

PROM: Prelabor rupture of membranes

**Supplementary Table 2. Summary of predictive performance across outcomes**

| **Outcome** | **C1** | **C2** | **Slope** | **AUC** |
| --- | --- | --- | --- | --- |
| Preterm birth | NS (p = 0.312) | NS (p = 0.089) | NS (p = 0.257) | 0.561 |
| PPROM | Trend (p = 0.055) | NS (p = 0.153) | **Significant (p = 0.030)** | 0.664 |
| PROM at term | NS (p = 0.562) | NS (p = 0.799) | NS (p = 0.454) | 0.549 |

C1: 1^st^ trimester cervical length

C2: 2^nd^ trimester cervical length

PPROM: Preterm Prelabor rupture of membranes

PROM: Prelabor rupture of membranes

**Supplementary Table 3. Adjusted ORs for PROM at term**

| **Variable** | **OR (95% CI)** | ***p* value** |
| --- | --- | --- |
| Maternal age (per year) | 1.00 (0.96-1.03) | 0.848 |
| BMI (per kg/m2) | 1.03 (0.99-1.06) | 0.156 |
| Smoking (yes vs no) | 0.51 (0.22-1.00) | 0.072 |
| GA at 2^nd^ measurement (per week) | 1.06 (0.89-1.25) | 0.524 |
| 2^nd^ trimester CL (per mm) | 0.99 (0.95-1.04) | 0.799 |
| Cervical slope (per mm/week) | 0.90 (0.68-1.19) | 0.454 |

OR: Odds ratio

BMI: Body mass index

GA: Gestational age

CL: Cervical length

**Supplementary Table 4. Sensitivity analyses: including progesterone users**

| **Variable** | **OR (95% CI)** | ***p*_value** |
| --- | --- | --- |
| Maternal age (per year) | 1.02 (0.99-1.05) | 0.220 |
| BMI (per kg/m2) | 1.02 (0.98-1.06) | 0.309 |
| Smoking (yes vs no) | 1.11 (0.58-1.96) | 0.725 |
| Antenatal progesterone | 5.93 (2.33-14.03) | **<0.001** |
| GA at 2^nd^ measurement (per week) | 1.02 (0.85-1.20) | 0.858 |
| 2^nd^ trimester CL (per mm) | 0.97 (0.92-1.01) | 0.125 |
| Cervical slope (per mm/week) | 1.15 (0.85-1.58) | 0.382 |

OR: Odds ratio

BMI: Body mass index

GA: Gestational age

CL: Cervical length

**Supplementary Table 5. Exploratory ORs in short cervix cohort**

| **Variable** | **OR (95% CI)** | ***p*_value** |
| --- | --- | --- |
| GA at 2^nd^ trimester | 0.64 (0.26-1.46) | 0.297 |
| Smoking (yes vs no) | 0.58 (0.07-3.23) | 0.560 |
| 1^st^ trimester CL (per mm) | 0.64 (0.31-1.10) | 0.152 |
| 2^nd^ trimester CL (per mm) | 1.36 (0.78-2.70) | 0.315 |
| Cervical slope (per mm/week) | 0.05 (0.00-4.80) | 0.245 |

OR: Odds ratio

BMI: Body mass index

GA: Gestational age

CL: Cervical length

# Supplementary Figures


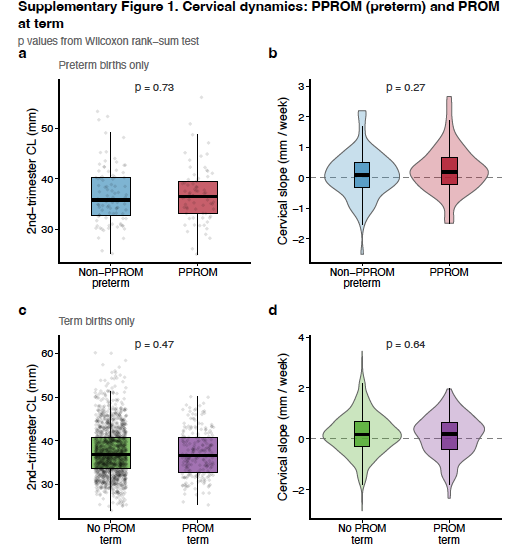


**Supplementary Figure 1.** Comparison of 2^nd^ trimester cervical length (CL) and cervical slope according to membrane rupture at labor onset. Panels (a) and (b) show 2^nd^ trimester CL (mm) and cervical slope (mm/week), respectively, in preterm births with and without preterm prelabor rupture of membranes (PPROM). Panels (c) and (d) show 2^nd^ trimester CL and cervical slope, respectively, in term births with and without prelabor rupture of membranes (PROM). No statistically significant differences were observed in second-trimester cervical length or cervical slope between women with and without PPROM or PROM. Wilcoxon *p* values are displayed.

**
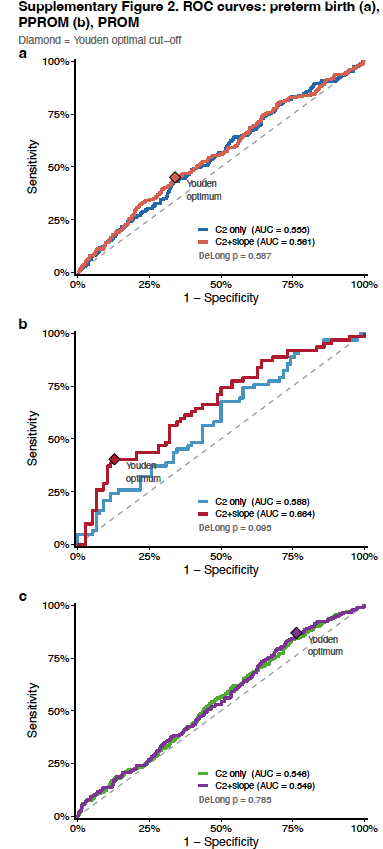
**

**Supplementary Figure 2.** Receiver-operating characteristic (ROC) curves comparing models including 2^nd^ trimester cervical length (C2) alone with models including C2 plus cervical slope. Panel (a) shows prediction of spontaneous preterm birth, panel (b) prediction of preterm prelabor rupture of membranes (PPROM), and panel (c) prediction of prelabor rupture of membranes (PROM) at term. The area under the curve (AUC) was low for preterm birth and PROM at term, and addition of cervical slope did not materially improve model discrimination. For PPROM, addition of cervical slope resulted in a modest increase in AUC, although the difference between models was not statistically significant. Diamonds indicate the Youden optimal cut-off, and DeLong *p* values are shown for comparison of the ROC curves.
